# Italian Chemists’ Contributions to Named Reactions in Organic Synthesis: An Historical Perspective †

**DOI:** 10.3390/molecules180910870

**Published:** 2013-09-04

**Authors:** Gianluca Papeo, Maurizio Pulici

**Affiliations:** 1 Department of Medicinal Chemistry, Nerviano Medical Sciences srl, Business Unit Oncology, Viale Pasteur 10, Nerviano 20014, MI, Italy; 2 Department of Chemical Core Technologies, Nerviano Medical Sciences srl, Business Unit Oncology, Viale Pasteur 10, Nerviano 20014, MI, Italy

**Keywords:** Italian chemists, named reactions, history of chemistry

## Abstract

From the second half of the 19th century up to modern times, the tremendous contribution of Italian chemists to the development of science resulted in the discovery of a number of innovative chemical transformations. These reactions were subsequently christened according to their inventors’ name and so entered into the organic chemistry portfolio of “named organic reactions”. As these discoveries were being conceived, massive social, political and geographical changes in these chemists’ homeland were also occurring. In this review, a brief survey of known (and some lesser known) named organic reactions discovered by Italian chemists, along with their historical contextualization, is presented.

## 1. Introduction

At some point in the history of organic chemistry, someone arbitrarily decided to christen a certain chemical transformation after its discoverer’s name. Arguably, this event might have occurred both to acknowledge the nominee’s merits and to render the complicated chemical jargon more usable. From that point on, organic chemistry started to possess a new phraseology, still peacefully coexisting with the rigorous IUPAC-approved chemical language. Evidence of this can easily be found by glancing at any laboratory bookshelf: a textbook containing “named organic reactions” or one with a similar title will surely be present, frequently as a second (or higher) edition [1,2,3,4]. Eavesdropping on chemists’ everyday working discussions is even more compelling: colleagues talking about a “Wittig reaction” surely outnumber those describing the “synthesis of an alkene, by reacting a given aldehyde with a suitable phosphorous ylide”. Mentors can be heard proposing to their students the use of the “Meerwein salt” to alkylate a given oxygen atom much more frequently than those proposing “triethyloxonium tetrafluoborate” instead. Finally, medicinal chemists trying to improve the results of their “three-component reaction between acetoacetate, a given aldehyde and urea” will be in the minority with respect to those who are in the process of improving a “Biginelli reaction”. Such examples are endless and so it is quite evident that the “named organic reactions” language encompasses a tremendous amount of embedded information. It represents a form of shorthand used to convey concepts that are otherwise much more complicated to exchange, with the additional advantage that it carries of “humanizing” chemistry, by recalling the original scientists standing behind the chemical transformations we are trying to exploit. However, the learning of this jargon, being two-step in nature, requires a double mnemonic effort. One must memorize the reaction and its discoverer’s name (which ideally entails also learning the correct pronunciation!). Additionally, the subjectivity of the nomination process renders reaction-naming an error-prone process. The overlooking of a seminal paper, the misattribution of credits (due to poorly diffused journals and their language), and the misrecognition of contributions from co-authors (often left out from the reaction name for the sake of simplicity) are examples of potential shortcomings. This subjectivity also renders complex any attempt at defining fixed criteria for whether a given reaction deserves to enter the hall of fame, or “Olympus” of named organic reactions. The boundaries of this Olympus were also somewhat fuzzy, being more or less confined either geographically or temporally. Thus, another crucial question is not why a chemical transformation is propelled from anonymity, but rather for how long it can withstand the changes of the rapidly evolving science of synthesis. However, apart from any epistemological considerations, the “named organic reactions” jargon still persists in enthralling the chemical community. As long as science uses it vividly, this language continuously grows. This, on one hand, will increase the efforts required to master it whereas, on the other, it will presumably continue to serve chemists in rapidly (and elegantly) exchanging ideas and solutions to their problems. In one word: to communicate. 

This special issue of the *Molecules* is dedicated to organic reactions discovered by Italian chemists. It is edited by Claudia Piutti, research chemist and grand granddaughter of Arnaldo Piutti, a renowned organic chemist of the early twentieth century, whose investigations on the relationships between stereochemistry and taste have been recently reviewed [5]. Considering the focus of this issue, we envisioned that a brief survey of some of the known and lesser known reactions named after their Italian discoverers may be useful for the reader interested in the topic. A short historical contextualization of these seminal contributions will hopefully render the reading more enjoyable. 

## 2. From the First Italian War of Independence (1848–1849) to the Proclamation of the Kingdom of Italy (1861)

At the dawn of the second half of the 19th century, the Italian peninsula persisted to be fragmented into a number of independent States extremely variable in size. Furthermore, the majority of the more economically developed territories (the Lombardy-Venetia Kingdom) was still in the hands of the Austrian-Hungarian crown. The increasing discomfort generated by this politically obsolete situation, as well as the growing sense of a national consciousness, culminated in the uprising of Palermo against the Bourbon monarchy (January 1848) and, two months later, the almost simultaneous rising of Milan and Venice against the Austrians (March 1848). A number of volunteers arriving from every part of Italy gathered around Carlo Alberto, the King of Piedmont who, in the meantime, had declared war on Austria. The troops fought courageously, but they were defeated by the superior Austrian Army first in Custoza, near Verona (June 1848), then in Novara (March 1849). Carlo Alberto abdicated in favor of his son Vittorio Emanuele II. The unfortunate epic of the first Italian war of independence formally ended. 

Like most of the intellectuals who lived during the pre-unification era, the chemists Raffaele Piria (1814–1865) and his student Cesare Bertagnini (1827–1857) were also patriots and were actively involved in the political life of their time. Working at the University of Pisa, they had joined that institutions’ volunteer corps and took part in the battle of Curtatone and Montanara (near Mantova), one of the episodes of the first war of independence. Stanislao Cannizzaro (1826–1910), another of Piria’s acolytes, who had at the time been on vacation in his native Sicily, took part instead in the revolution that broke out on the island against the Bourbons.

Professionally, the years spent at the University of Pisa were particularly fruitful for Piria, undoubtedly one of the most prominent scientists of his time who is remembered for his numerous contributions to organic chemistry [6]. In fact Piria published his seminal papers during his professorship at the local University. 

**Scheme 1:**
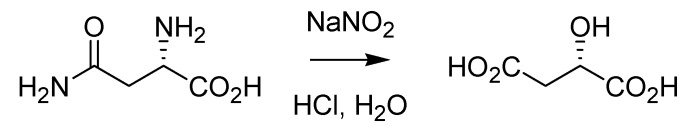
The Piria diazotization reaction (1846).

Interestingly, what was referred to as the Piria reaction until a few years ago was the transformation of primary amines and/or amides to the corresponding alcohols and/or carboxylic acids by means of nitrous acid (Scheme 1). This reaction, which is nowadays known to proceed with retention of configuration, was discovered by Piria while working on asparagine and described in 1846 [7] and has retained a certain analytical value for the detection of amino- groups for quite a long time.

**Scheme 2:**
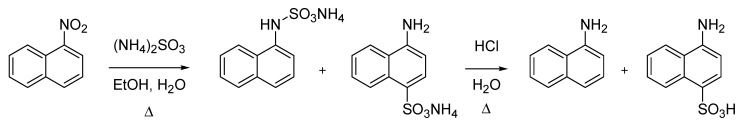
The Piria aminosulfonic acid synthesis (1851).

However, what is generally acknowledged as the Piria reaction is the reduction of an aromatic nitro compound by means of a sulfite delivering a mixture of sulfamic- and aminosulfonic acid salts, the former being hydrolyzed to the corresponding amine upon acidic treatment [8,9]. The reaction was originally described on 1-nitronaphthalene (Scheme 2).

In the context of this review, it is worth mentioning that both the reduction of carboxylic acids to aldehydes in the presence of calcium formate and the thermal decarboxylative coupling of carboxylic acid salts to form the corresponding symmetric ketones were formerly referred to as the Piria reaction(s) [10].

**Scheme 3:**
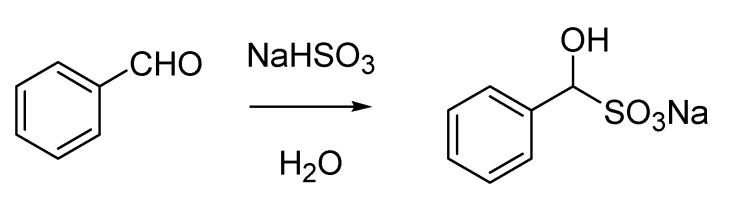
“Bertagnini’s salt” (1851).

Cesare Bertagnini, one of Piria’s favorite students inherited his Chair at the University of Pisa when his mentor moved to Turin. He is also remembered thanks to some outstanding work he published during his extremely short life. His name is associated with the formation of the so called Bertagnini’s salt, the adduct between an aldehyde and an alkaline bisulfite (Scheme 3). This procedure was routinely used in the past to purify aldehydes and was first described by Bertagnini in 1851 [11,12,13].

**Scheme 4:**
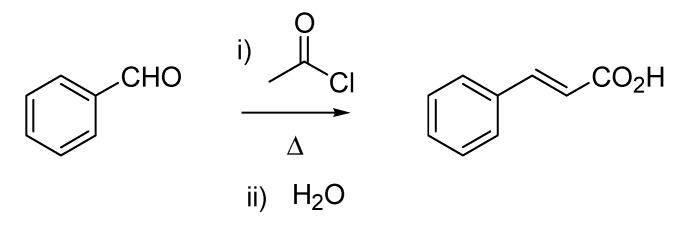
The Bertagnini reaction (1856) later modified by Perkin (1868).

Bertagnini’s name is also linked to the Bertagnini-Perkin reaction. Although this carbon-carbon bond forming transformation is mainly reported as the Perkin reaction (the condensation between acetic anhydride and benzaldehyde delivering cinnamic acid), described by the English chemist as early as 1868 [14], the same conversion was anticipated by Bertagnini [15,16], who used acetyl chloride instead of acetic anhydride (Scheme 4). Additionally, the twosome Chiozza-Bertagnini is also found in references associated with the same type of transformation, thus recognizing, in a perhaps questionable form of parochialism, the contribution of Luigi Chiozza (1828–1889), who had conducted prior work on the synthesis of cinnamaldehyde [17].

**Scheme 5:**
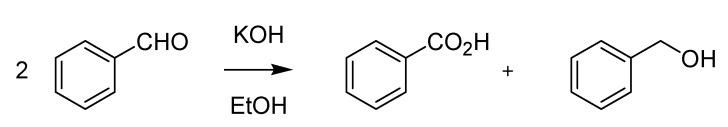
The Cannizzaro reaction (1853).

On the contrary, the name of Cannizzaro has come down to us more clearly in association with a specific organic reaction, the base-mediated disproportionation of a non-enolizable aldehyde into the corresponding alcohol and carboxylic acid. The original reaction that he described while he was at the Collegio Nazionale di Alessandria (Piedmont) in 1853 [18], was performed using bitter almond oil (benzaldehyde) and potash as the base (Scheme 5).

In the meantime and despite the unhappy ending of the first Italian war of independence, the will to unify Italy was still smoldering under the ashes. At the beginning of 1859, Vittorio Emanuele II and his prime minister, the Count of Cavour, made a secret alliance with Napoleon III, the emperor of France, as they realized that there was no hope for Piedmont to defeat the Austrians alone. The key point of this alliance treaty was that France formally bound itself in helping Piedmont in case of aggression by the Austrians, as did in fact occur in April of that year. The war that followed (the second Italian war of independence) was rapidly won by the France-Piedmont allied troops and an armistice was signed in July. As a result, the vast majority of Lombardy was ceded to the King of Piedmont, while the Venetian region still remained part of the Austrian empire. In few months, with the consent of England and France, most of the population of central Italy was allowed to express their will to join the now enlarged Piedmont Kingdom. It was a complete success, but Unification of the country was still far from complete, with the Bourbon-controlled southern half of the Italian peninsula representing the next major obstacle to overcome. It took another year for the “red shirts”, a volunteer army led by the warlord and politician Giuseppe Garibaldi, to occupy Sicily and turn towards North, finally defeating the Bourbons close to the Volturno River, north-west of Naples. It was October the 2^nd^, 1860.

While these dramatic events were taking place, another struggle, led by the Italian chemist Stanislao Cannizzaro, was occurring on scientific ground. In September 1860 the first international meeting of chemists, called by Kekulé, was held in Karlsruhe, Germany. Through his masterpiece “Sunto di un corso di filosofia chimica” (sketch of a course of chemical philosophy) [19] which he distributed at the very end of the conference, Cannizzaro successfully reiterated the hypothesis of Avogadro (Avogadro himself, was also, by the way, an Italian physical chemist), that certain gaseous elements possess diatomic molecules, thus requiring the doubling of their previously considered atomic weights. 

**Figure 1:**
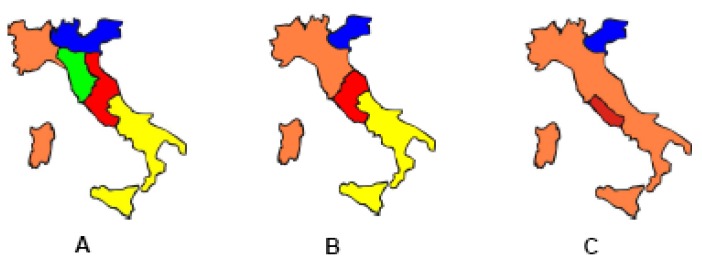
Maps of Italy in 1859 (**A**), 1860 (**B**) and 1861 (**C**).

On 17th March 1861 the Kingdom of Italy was proclaimed. However, the unification process was far from being truly accomplished. Apart from the Venetian region, there was still a significant chess-piece remaining on the board: Rome (Figure 1). 

## 3. From Florence as the New Capital of Italy (1865) to the Assassination of King Umberto I (1900)

Following an agreement with Napoleon III, in June 1865 Italy moved its capital from Turin to Florence. One year later, an alliance with Prussia against the Austrians was signed, under the terms of which Italy would have gained rights to the Venetian region. War against Austria (known as the third war of independence) was shortly declared and quickly won, mainly due to the superiority of the Prussian army. The peace treaty was ratified in October 1866, and following a referendum, the Venetian region was annexed to the Kingdom of Italy. However, a large portion of the northeast territories still remained in the hands of the Austrians: the Trentino and Venezia-Giulia regions. The inhabitants of those lands would have to wait for the end of WWI before being recognized as Italian citizens. 

**Figure 2:**
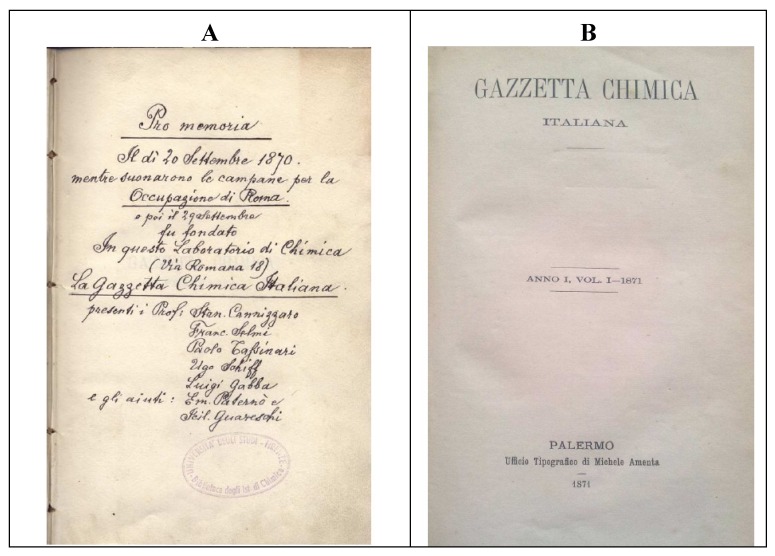
The founding act of the Gazzetta Chimica Italiana (Italian Chemical Gazette) (29 September 1870, **A**) and the frontispiece of its first issue (31 March 1871, **B**).

The long-awaited opportunity for Rome’s incorporation into the Italian Kingdom was presented by the Franco-Prussian war of 1870. Forced to withdraw its garrison from the city to reinforce its army and later defeated at Sedan by the Prussians, France was no longer in a position to protect Rome and thus Italian troops entered the city on 20th September of that year. Nine days later, “while the bells were ringing to celebrate the occupation of Rome”, seven of the country’s most prominent chemists, headed by Cannizzaro, met in Florence and founded the earliest Italian chemical journal, the “Gazzetta Chimica Italiana” (Italian Chemical Gazette) (Figure 2A), whose first issue was published on March 31st, 1871 (Figure 2B). In the meantime, a further referendum had ratified the annexation of Rome, which shortly became the capital of the Kingdom. 

**Scheme 6:**
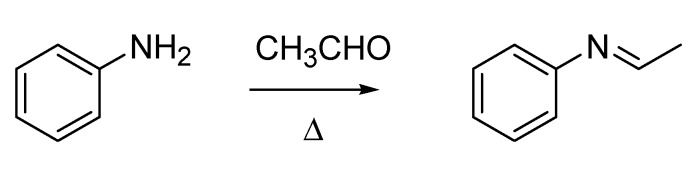
The Schiff base synthesis (1864).

Italian chemistry of this period has another “giant” to whom recognition is due in the context of named organic reactions, *i.e.*, Hugo (often Italianized as Ugo) Schiff (1834–1915). Although German by birth, Schiff spent most of his life and career in Italy, and he was one of the “Magnificent Seven” subscribers of the founding act of the Gazzetta Chimica Italiana (Figure 2A). He was at the University of Pisa when he published his first paper on a “new type of organic base” [20], the compounds that still today are called the Schiff bases (Scheme 6). The work describing the Schiff fuchsin aldehyde test [21], of paramount importance for staining organic aldehydes in biological samples, was also published in that period.

At the beginning of 1878, King Vittorio Emanuele II died. The throne was inherited by his son Umberto I who signed the Triple Alliance Treaty with Austria and Germany in 1882. This treaty forged an agreement of mutual military support in case of aggression by any other power, while a neutral behavior would be adopted in case of aggressive actions against others undertaken by any one of the signatories. The same year Giuseppe Garibaldi, the true military leader behind the unification of Italy, died. Desiring to be considered on an equal footing with the most advanced European nations, Italy started its own colonization campaign in Africa in 1885, by disembarking its troops in Massawa (Eritrea). Efforts to further expand its domination to Ethiopian territories culminated in 1887 with the massacre of Dogali where nearly all of the 500 Italian soldiers were killed after having fought against overwhelming forces. That same year the Triple Alliance Treaty was renewed. In 1892 one of the most outstanding figures of Italian politics, Giovanni Giolitti (1842–1928), was nominated Prime Minister. Giolitti was a left-wing liberal and is still the second-longest serving Prime Minister in the history of Italy after the dictator Benito Mussolini (1883–1945). During Giolitti’s first mandate (1892–1893), the Socialist Party of Italian Workers was founded, and the ensuing years saw the establishment of the first association of Italian chemists, the Chemical Society of Milan (1895), followed by Turin’s Industrial Chemistry Association (1899). 

The Italian colonial adventure in Africa was reawakened in 1894. After a couple of modest military victories, the attempts to invade Ethiopia were finally frustrated first at Amba Alagi then at Adwa (1896) by the soldiers of Menelik II, the Ethiopians’ Emperor. A peace treaty was ratified at Addis Ababa at the end of the year. Accordingly, the Italian colonial Empire would be limited to Eritrea and Somalia. The cost of these war operations exacerbated an already difficult economic crisis. A number of meetings, demonstrations and strikes occurred throughout the Nation. In May 1898, a massive strike in Milan culminated in a massacre by the military forces present. It was during this highly agitated social climate that Gaetano Bresci, a Tuscan anarchist who had emigrated to the US, returned to Italy with the intention of assassinating the monarch to successfully accomplish his mission. On July 29, 1900 Bresci killed King Umberto I in Monza (near Milan), shooting him four times while he was attending a sports meeting. 

**Scheme 7:**
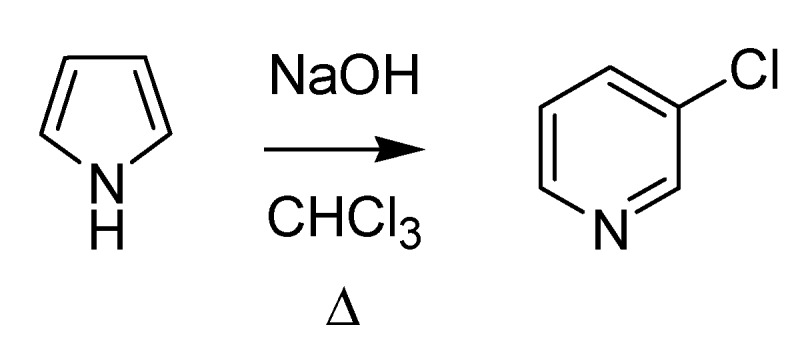
The Ciamician-Dennstedt rearrangement (1881).

On the scientific front during this period, in 1881 Giacomo Ciamician (1857–1922), at that time Cannizzaro’s assistant at the University of Rome, co-authored together with Dennstedt a paper [22,23] that allowed also him to enter the Olympus of named reactions for the first time. The Ciamician-Dennstedt rearrangement entails expansion of the pyrrole ring to form a pyridine derivative by means of chloroform (or other halogeno- compounds) and a base through the *in situ* generation of dichlorocarbene (Scheme 7).

**Figure 3:**
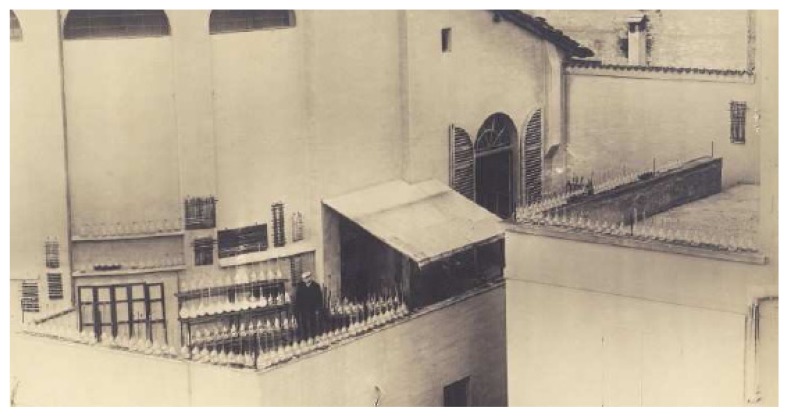
The dawn of photochemistry. Ciamician surrounded by a number of sun-exposed flasks on his laboratory terrace at the University of Bologna.

Ciamician’s interest in pyrrole chemistry dated back to the years when he was a student, first in Vienna and then in Giessen, but persisted throughout his highly productive career. From pyrrole, through to plant chemistry, he gradually came upon the world of photochemistry, where he made a ground-breaking contribution through the discovery of several key reactions and is today unanimously recognized as a founding father of this branch of chemistry (Figure 3). 

**Scheme 8:**
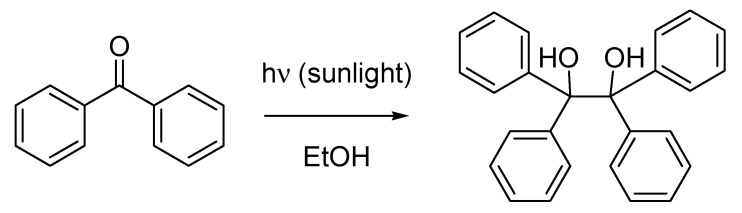
The Ciamician photocoupling (1900).

The remarkable scope and impact of Ciamician’s work in the field of photochemistry can be perceived in the ambiguity with which the eponymous photochemical reaction has variously been described. According to most current textbooks for example [1], the Ciamician reaction consists in the reductive photocoupling of ketones leading to the formation of 1,2-diols [24] (Scheme 8).

However, during the golden age of photochemistry the Ciamician reaction was also proposed [25] as being the intramolecular [2 + 2] cycloaddition of alkenes, which Ciamician and his co-worker Paul (Paolo) Silber had described in reference to the formation of a camphor derivative during prolonged sunlight exposure of carvone [26]. Yet again, according to Giovanni Battista Bonino, who later wrote the entry for Giacomo Ciamician which appears in the authoritative Treccani Encyclopedia (considered in Italy to be the definitive source of reference for information) [27], the Ciamician reaction is the photochemical disproportionation of nitrobenzaldehyde to nitrosobenzoic acid, another significant transformation described by Ciamician and Silber [28].

**Scheme 9:**
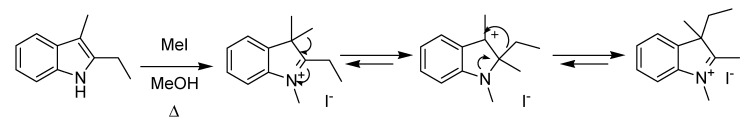
The Plancher rearrangement (1898).

These contributions reflect the profound interest and faith Ciamician had in photochemistry and its potential applications, which are highlighted in his famous dissertation on “the future of photochemistry” of 1912 [29], where he advocated the use of sunlight to produce energy and predicted the modern utilization of solar cells and can thus also be considered a pioneer of “green chemistry”. In Bologna, the University where he spent most of his career, he was the founder of a school of Chemistry where many outstanding scientists were educated, and which still stands today. One of his earlier fellows, Giuseppe Plancher (1870–1929), is associated with a named reaction published before the end of the century. The Plancher rearrangement (sometimes referred to as the Plancher-Ciamician rearrangement) [30] was discovered by the reaction of 2-ethyl-3-methylindole with methyl iodide, leading to 1,2,3-trimethyl-3-ethyl indolenium iodide, which was later explained by a Wagner-Meerwein type migration of alkyl groups from position 3 to position 2 of an indoleninium species (Scheme 9). Since the migration reactions are in equilibrium, the process eventually leads to the most thermodynamically stable isomer. 

**Scheme 10:**
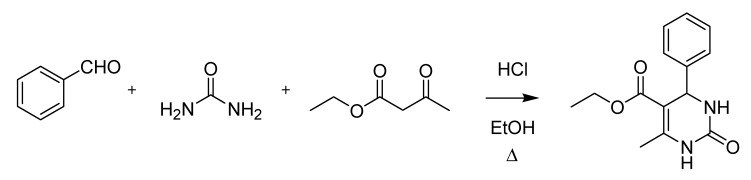
The Biginelli pyrimidine synthesis (1891).

The last decade of the nineteenth century witnessed several important Italian contributions to organic chemistry. Pietro Biginelli (1860–1937) published the first accounts of his famous pyrimidine synthesis in 1891 (Scheme 10), soon after joining the laboratory of Hugo Schiff in Florence [31,32]. 

Biginelli, whose scientific activity culminated in Rome at the “Istituto Superiore di Sanità” (State Medicinal Institute) where he had the opportunity to develop an analytical method also known as the Biginelli test [33], soon decided to turn to a non-academic career [34]. His involvement in chemical research lasted a little more than a decade, but it was very intense, and left a well-defined mark. Arguably, this can be attributed to the education he received while he was a student in Turin, where his mentor was the founder of another important regional school of chemistry: Icilio Guareschi (1847–1918).

**Scheme 11:**
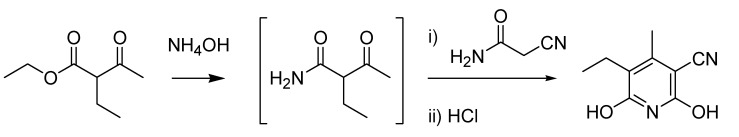
The Guareschi reaction (1896).

Icilio Guareschi stands out for being a complex figure of man, both scientific divulgator and chemist. In 1866, while still a high school student in Parma, he participated as a volunteer in the third war of independence against the Austria-Hungary Empire. Later he became an active pacifist. He produced an impressive collection of works, encompassing dozens of publications devoted to chemical education, including a 13-volume encyclopedia dedicated to applied chemistry, unofficially known as “the Guareschi” [35], and to history of chemistry and science in general. The work that ensured him a place in the list of named organic reactions was published in 1896, while he already was a well-known professor at the University of Turin. The Guareschi reaction [36], which is also sometimes referred to as the Guareschi-Thorpe [37] reaction, deals with the synthesis of pyridines by condensation of cyanoacetic ester or primary amide with acetoacetic ester in the presence of ammonia (Scheme 11).

**Scheme 12:**
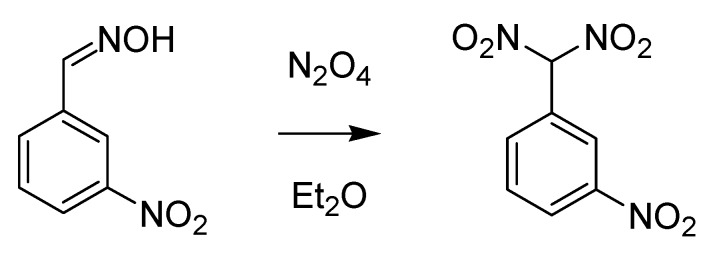
The Ponzio reaction (1897).

Another chemist from the University of Turin who was later acknowledged thanks to the intense work he carried out on dioximes is Giacomo Ponzio (1870–1945). The reaction he published in 1897 on the oxidation of aldoxime by means of nitrogen dioxide in ether [38] is today known as the Ponzio reaction (Scheme 12). 

**Scheme 13:**
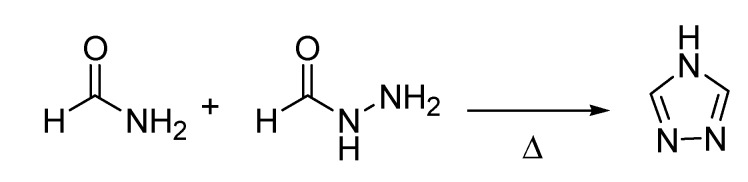
The Pellizzari reaction (1894).

A few years earlier, in 1894, another chemical transformation that would later to be referred to as a named reaction, had appeared in the literature. This is the Pellizzari reaction [39], after Guido Pellizzari (1858–1938) a professor of medicinal chemistry at the University of Florence and a former fellow of Hugo Schiff, who disclosed a new method for the synthesis of 1,2,4-triazoles (Scheme 13). 

**Scheme 14:**
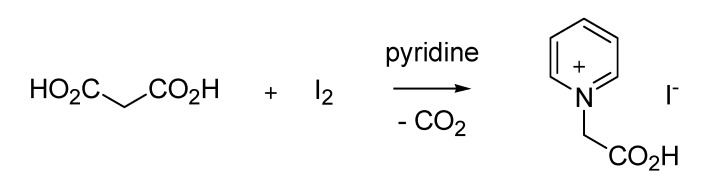
The Ortoleva-King reaction (1900).

After his degrees in pharmacy and chemistry and before becoming an appreciated high-school teacher in Milan, Giovanni Ortoleva (1868–1939) worked as a pharmaceutical chemistry researcher at the University of Palermo. During this interlude he synthesized β-iodocinnamic acid by reacting cinnamic acid with elemental iodine in the presence of pyridine [40]. While further investigating the scope of this reaction, he then employed malonic acid as a substrate, which unexpectedly delivered a pyridinium betaine with loss of carbon dioxide [41]. This posed the basis for a novel and general transformation that encompasses the formation of *N*-alkyl pyridinium derivatives of activated methyl and methylene species, which in turn find a number of synthetic applications [2]. This reaction became later known as the Ortoleva-King reaction (Scheme 14) by acknowledging the contribution of L. Carroll King from Northwestern University, Illinois, who in 1944 further expanded the scope of the reaction [42].

**Scheme 15:**
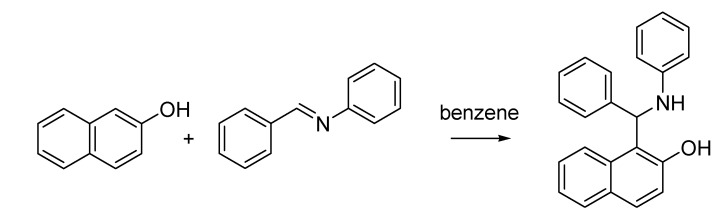
The Betti reaction (1900).

The Italian contribution to the chemistry of the last decade of the 19th century closed with another publication that appeared in 1900 by Mario Betti (1875–1942), a young graduate from the University of Pisa working in Schiff’s laboratory in Florence. The Betti reaction [43] is nowadays considered as a multicomponent condensation between a phenol, an aldehyde and an aromatic amine that produces α-aminomethylphenols, though in the original paper the author described the use of a preformed imine (Scheme 15). This can be regarded as a special case of the more general (and subsequently discovered) Mannich reaction.

## 4. From the Beginning of the 20th Century to the Outbreak of the Great War

After the death of Umberto I, the crown was inherited by his son Vittorio Emanuele, who took the throne as Vittorio Emanuele III. He was the penultimate king of Italy. The beginning of the 20th century was one of the most economically rewarding periods for the Country. The national income doubled and savings increased four times. Italian foreign commerce was healthier than that of Germany and England. It was during these prosperous days, christened throughout Europe as the Belle Époque, that the Chemical Society of Rome was founded in 1902, once again thanks to the initiative of Cannizzaro, who since 1871 had been appointed Professor in Rome since, as well as one of his most talented disciples, the Marquis Emanuele Paternò. Seven years later, this Society merged with the that of Milan, giving birth to the “Società Chimica Italiana” (Italian Chemical Society), which is still in place today and which currently has more than 3,500 members. During Giolitti’s fourth tenure as Prime Minister (1911–1914) a profound reform of education occurred, which raised the minimum age of compulsory education to twelve and which transferred management of the schools from city administrations to the State. A further crucial step towards the modernization of Italy was accomplished in June 1912 with extension of the right of vote to all 30-year-old male citizens, thus increasing the politically active population from 7% to more than 23%. 

**Figure 4:**
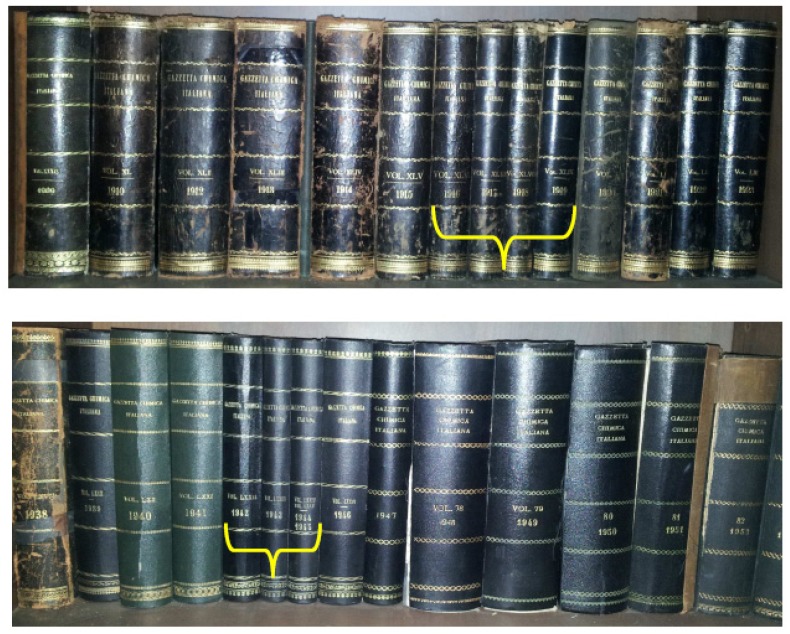
The negative impact of war on scientific publications. Note the smaller size of the Gazzetta Chimica Italiana volumes between 1916 and 1919 (top) and between 1942 and 1945 (bottom) (from the Nerviano Medical Sciences library).

However, an economic recession was approaching. As a consequence, peace became as volatile as ether. To distract popular opinion from the incoming storm and after a secret agreement with France, the Italian government decided to enlarge its colonial dominions. In 1911 troops invaded the Ottoman territories of Tripolitania and Cyrenaica (now parts of Libya), forced the Dardanelles strait and obliged Turkey to recognize (in October 1912) Italian sovereignty over the conquered African lands. But this was just a skirmish. On May 24, 1915, after nearly one year of neutrality from the very beginning of the war that would stain Europe and the rest of the world with blood for more than four years, Italy joined the Triple Entente (United Kingdom, France and Russia) and entered into the conflict against the Central Powers (Austria-Hungary and Germany). It was the Great War, which would sadly be recorded in historical textbooks as the First World War (Figure 4, top). 

**Scheme 16:**
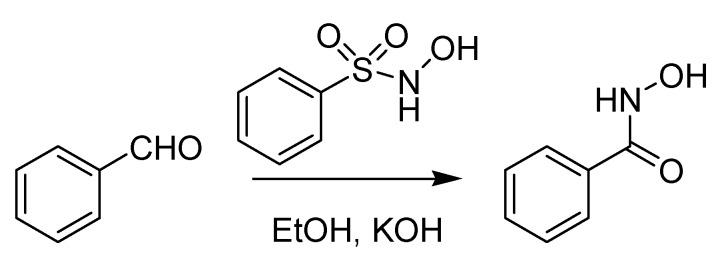
The Angeli-Rimini reaction (1901).

The first Italian named reaction of the 20th century has its roots back in 1896, when Angelo Angeli (1864–1931), an outstanding scientist who would later be nominated several times for the Nobel prize [44], published a paper describing the preparation of the sodium salt of nitro hydroxylamine (Na_2_N_2_O_3_) [45]. This compound, known as Angeli’s salt, is endowed with several pharmacological properties stemming from its instability, which leads to the formation of nitrous acid and nitroxyl (HNO) [44]. Subsequently, Angeli [46] noted that HNO could be “fixed” by aldehydes, forming hydroxamic acids. It was however Enrico Rimini (1874–1917), one of Ciamician’s numerous students together with Angeli, who in 1901 disclosed a more practical version of this reaction, that he employed to detect aldehydes [47]. The reaction, using N-hydroxybenzensulfonamide (Piloty’s acid) rather than the Angeli’s salt as a donor of nitroxyl, is devoid of the interference of nitrous acid and was later christened with the name of both scientists. The Angeli-Rimini reaction (Scheme 16) has both analytical (for the identification of aldehydes) and preparative value (for the synthesis of hydroxamic acids).

It is worth mentioning that the name of Enrico Rimini is often associated with another analytical method, the so-called Rimini-Schryver reaction [48]. This test is used to quantitatively estimate allantoin in biological fluids, and is based on a procedure developed by Rimini for the detection of formaldehyde [49] later implemented by Schryver [50].

**Scheme 17:**
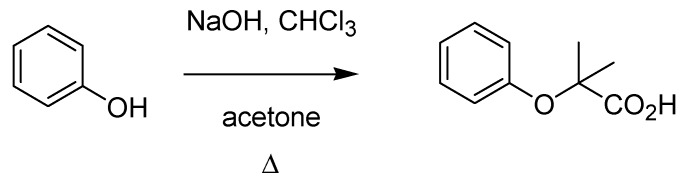
The Bargellini reaction (1906).

Guido Bargellini (1879–1963) was a brilliant chemist who, after a post-doctoral experience in Emil Fischer’s laboratory, was appointed at the University of Rome, where he spent most of his career. His interests in coumarins prompted Bargellini to investigate a multicomponent reaction between phenol, chloroform and acetone in the presence of alkali previously described in a German patent [51], thus finding that the structure attributed to the product was misassigned. He discovered that the reaction produced a carboxylic acid (instead of a phenol as originally proposed) [52] (Scheme 17), thus paving the way for a new transformation, the Bargellini reaction, whose number of variations contributed to its wide application both in organic and medicinal chemistry.

Bargellini represents a sort of joining link between the classical era of Italian chemistry and the modern times. While from an anagraphical point of view he is projected towards the latter, he is however bound to the former by his direct link with two great masters: Cannizzaro, who first called him to Rome, and Cannizzaro’s successor at the chair of general chemistry of the same University: Emanuele Paternò.

**Scheme 18:**
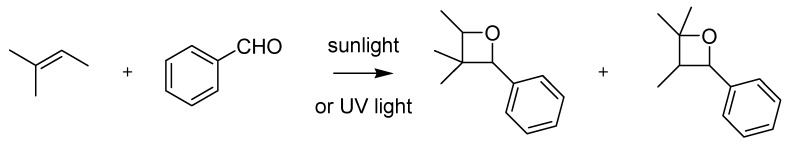
The Paternò-Büchi [2 + 2] cycloaddition (1909).

Emanuele Paternò (1847–1935), born Marquis of Sessa, was another prominent scientific personality who was also actively involved in the political life of his native homeland. As already mentioned (see Section 2), he was one of the founders and the first director (from 1870 to 1919) of the Gazzetta Chimica Italiana. He served on many other fronts as well, being for instance nominated Dean at the University of Palermo and Senator of the Kingdom. As a chemist, he is often associated with the pioneering studies on tetrahedral carbon and on cryoscopy. In organic synthesis, he studied the photochemical cycloaddition of olefins to carbonyl compounds leading to oxetanes [53]. Paternò published his results about this reaction in 1909, but he had no means to establish its regiochemical outcome. It was only 45 years later that Büchi, by capitalizing on spectroscopic experiments, discovered that the reaction can be regio- and stereoselective depending upon the nature of the reactants [54], thus providing a fundamental piece of information of what is nowadays called the Paternò-Büchi reaction (Scheme 18).

## 5. Italy between the Wars

Italy fought WWI chiefly on the “Italian front” (the northeast of the Country) against the Austrian-Hungarian troops. It was essentially a war of position, with a number of tactically useless assaults and counterassaults on a difficult mountainous terrain, which meaninglessly moved the front at a high cost in human lives. After nearly 2 years and a half of stagnation, the Italian army was defeated at Kobarid (Caporetto in Italian, now in Slovenia) in October 1917. However, the Austrians did not capitalize on this success. The centrifugal forces that were acting within the Austrian-Hungarian empire due to claims for independence of its constituting nations contributed to the dissolution of its multiethnic army. The decisive battles close to the river Piave and the town of Vittorio Veneto (near Treviso, Venetian region) allowed the Italians to victoriously conclude the war. The armistice between Italy and Austria was formally signed on November 3, 1918 at Villa Giusti (Padua). As a consequence, Trentino and Venezia-Giulia territories became part of the Kingdom. However, nationalists were still not satisfied. The annexation of the largely Italian-speaking Dalmatian region and the city of Rijeka (Fiume in Italian, now in the State of Croatia) to Italy was denied by the other WWI co-winners gathered at Versailles (France). The already tense political and economical situation arising from the post-war crisis was further exacerbated by the socialists who systematically and often violently, fomented public opinion. During the following year (1919) several significant events occurred. Benito Mussolini, a prior left-wing journalist, founded the “Fasci Italiani di Combattimento” (Italian Groups of Combat), an organization which recruited a significant number of followers, wary of the rise of the socialism (with which Mussolini himself had previously flirted). In addition, the Popular Party (the future Christian Democrats) had its first meeting, and the right of vote was granted to all 21-year-old (and older) male citizens. In September the poet, novelist and swashbuckler Gabriele D’Annunzio and his legionnaires occupied the Croatian city of Rijeka, which they held until they were forced to leave by the Italian troops in December 1920. Six months earlier Giolitti had become prime minister for his fifth (and last) time. In January 1921 the left wing of the socialistic movement founded its own party: the Italian Communist Party. During these agitated years, Italy was poisoned by a number of violent strives between the different political factions. None of these was able to build up a coalition in parliament strong enough to withstand the ascent of Mussolini’s fascists, who formed a fully-fledged political party in November 1921 and nearly one year later its members (the so-called “black shirts”) occupied Rome. Vittorio Emanuele III had little choice. He asked Benito Mussolini to form the new government, leaving the stage to the darkest period in the history of the Country. 

**Scheme 19:**
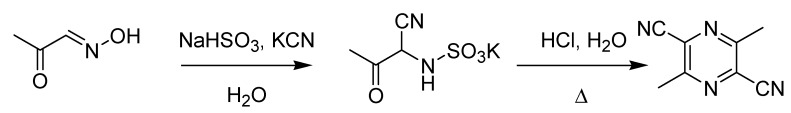
The Gastaldi pyrazine synthesis (1921).

One of the most talented chemists during the interlude between the wars was Carlo Gastaldi (1884–1962), a knowledgeable professor of chemistry who spent his earlier and later career in Sassari (Sardinia). In 1916 he had just joined the University of Turin, when he was drafted into army and sent to the front. After spending three years at the army’s “pyrotechnic laboratory”, he went back to the laboratory directed by Ponzio, and continued the research that culminated with the publication of his pyrazine synthesis [55] (Scheme 19). 

**Scheme 20:**
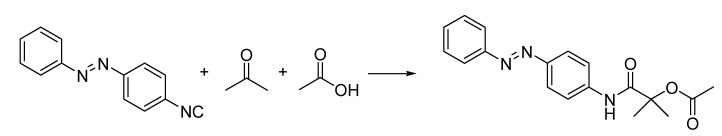
The Passerini reaction (1921).

During the course of 1921 another publication appeared in the Gazzetta Chimica Italiana for which his author would later be remembered for the eponymous reaction [56,57]. Its author, Mario (Torquato) Passerini (1891–1962), had also taken part in the war while still a student. Passerini had graduated from the University of Florence in 1916 during army leave and after completing military service he worked at the same University for several years. The Passerini reaction is a multicomponent reaction where an isonitrile, a carboxylic acid and a carbonyl compound are reacted in an apolar solvent leading to a α-acyloxyamide (Scheme 20). Its publication, fruit of the scientist’s Florentine years, provided the first important account on the chemistry of isonitriles, and is undoubtedly one of the most cited Italian named reactions. 

The treaty of Lausanne (July 1923), which definitively recognized Italian sovereignty over the Dodecanese islands (now part of Greece) and the re-annexation of Rijeka, formalized in January 1924, increased the government’s consensus, up to the point at which a few months later (June 1924), it brazenly commissioned the murder of Giacomo Matteotti, a young socialist leader and a dictatorial regime soon followed in January 1925. Mussolini was subsequently the target of several assassination attempts, giving him the pretext to render illegal all opposition parties and newspapers, and to reintroduce the death penalty that had been banned since 1889. Aiming at a positive resolution of the conflict between the Italian Kingdom and the Catholic Church dating back to the annexation of Rome, Mussolini signed the Lateran Treaty in 1929, a formal reconciliation with the Vatican. Later the same year, the Wall Street crash sparked the deepest global economic crisis ever experienced. 

**Scheme 21:**
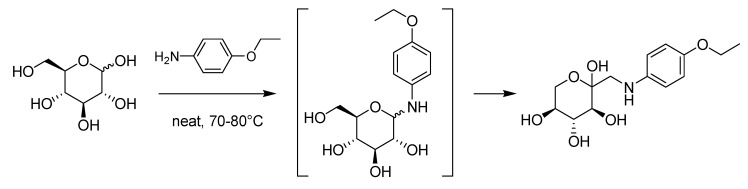
The Amadori rearrangement (1925).

During this politically and economically turmoiled period, Mario Amadori (1886–1941), originally at the University of Padua and subsequently at the University of Modena, performed his research on sugars [58] discovering what nowadays is referred to as the Amadori rearrangement. This transformation involves the reaction of an aldose with a suitable amine leading to the corresponding glycosylamine which in turn rearranges to the corresponding ketoseamine (Scheme 21).

In June 1934 Mussolini and Hitler, the leader of the Nazi party and Chancellor of Germany, met for the first time. In pursuing his dream of an Italian “Empire” and despite the economical and financial sanctions inflicted by the League of Nations, Mussolini invaded Ethiopia in 1935. After having re-conquered Adwa, the Italian troops finally entered the capital Addis-Ababa in May 1936. It was the “magic moment” of the fascist era.

**Scheme 22:**
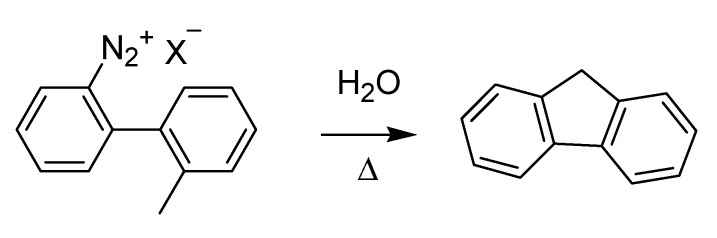
The Mascarelli fluorene synthesis (1936).

In the same year, a novel method for the synthesis of fluorene via the formation of an intermediate diazonium ion was published [59] (Scheme 22). The method is known as the Mascarelli fluorene synthesis after its discoverer, Luigi Mascarelli (1877–1941), who grew up at the school of Ciamician in Bologna and later inherited the chair of Guareschi at the University of Turin.

1936 also saw outbreak of the Spanish civil war, in which Italy supported the Nationalist front headed by the general Francisco Franco against the pro-government Republicans by providing Franco with a military contingent. This volunteer army heavily contributed to Franco’s seizure of power (April 1939) who established a fascist-like dictatorship. Aiming at being a protagonist of the political situation in Europe and not just a background actor, Mussolini aped Hitler by introducing in 1938 the racial laws against Jews. In April 1939 the Italian troops occupied Albania. With the invasion of Poland by the Nazis (1 September 1939) and the consequent declaration of war by France and England against Germany, the Second World War officially commenced. Erroneously convinced that the war would end swiftly with a German triumph, Mussolini entered the conflict at Hitler’s side in June 1940. At the time, although many Italians realized how inopportune this act was, few imagined the immense catastrophe the country would undergo.

## 6. WWII Aftermaths and the Contemporary Times

Italy entered WWII with a technically and organizationally unprepared army. After some useless skirmishes with the French, occurring just few days before France was forced to sign the armistice with Germany, and a handful of modest successes in northern and eastern Africa, the Italian troops headed towards a misfortunate fate on every front they fought. The futile invasion of Greece (started in October 1940) was disastrously conducted and only the arrival of the Germans allowed the Italians to not to be completely defeated. Joint German and Italian forces were subsequently driven out of Africa (May 1943) and their advance inside the Russian territories ended with the dramatic full retreat of the ARMIR (“Armata Italiana in Russia”, Italian Army in Russia) and the German defeat at Stalingrad (January 1943, the bloodiest battle in history). In July 1943 the Anglo-American troops (US entered the conflict in December 1941) disembarked in Sicily. They encountered a feeble resistance by the Italians. While the Allied army relentlessly continued its march up North, everybody realized that the Fascist age was close to the end. On 25 July 1943 the “Gran Consiglio del Fascismo” (Grand Council of Fascism), the main body of the fascist government, voted the deposition of Mussolini, who was suddenly arrested and replaced by Marshal Badoglio, the signer of the unconditional armistice between Italy and the Allied forces that would occur on 8 September 1943. Immediately thereafter, Badoglio and the King Vittorio Emanuele III left the Capital to settle the new government in the South of Italy. Few days later, the now enemy German troops occupied Rome and then liberated Mussolini from his confinement. Supported by the Nazis, the Italian dictator founded the “Repubblica Sociale Italiana” (Italian Social Republic) with jurisdiction over the North of the Country up to Rome (the part of Italy still in Germans’ hands). It was a hopeless, pathetic attempt to reinstall the fascist regime. While most of the opponents entered the partisan brigades to fight the fascists in what was a true civil war, the Nazis were defeated by the Allied army both in France and in Italy, where an armistice was signed on 29 April 1945. In February the same year, the Allied heads of state had met in Yalta (Crimea) to define the political and geographical asset of post-war Europe. They had also ratified the project concerning the creation of the United Nations. The insurrection of the partisans in Milan (25 April), and the execution of Mussolini (28 April) two days before the suicide of Hitler in his bunker in an already Russian-occupied Berlin, led to the end of the WWII in Europe. The unconditional surrender was signed by the Germans on 8 May 1945. It would take four extra months of war and two atomic bombs on Hiroshima and Nagasaki to force also Japan (that had entered the conflict in December 1941) to surrender (2 September 1945). The biggest human tragedy of the modern era was finally ended. Back from Auschwitz, the sadly famous Nazi concentration camp, Primo Levi, an Italian Jewish chemist and writer, collected his memoires of lager survivor in the book “Se questo è un uomo” (If this is a man), a literary masterpiece that should be present in everyone’s library. 

In May 1946, the King Vittorio Emanuele III abdicated in favor of his son Umberto, who took the throne as Umberto II. However his Kingdom lasted roughly a month: on 2 June 1946 a constitutional referendum set forth the birth of the Italian Republic. Umberto II went into exile in Portugal, where he died in 1983. 

The first president of the newborn Republic (Enrico de Nicola, a member of the Italian Liberal Party) was elected at the end of June 1946. One and a half years later, in December 1947, the Constitution of the Italian Republic, the chief legislative act ruling the general principles on which the Republic itself is founded, was enacted. In the late ‘40s Italy started its economic resurgence, heavily supported by the US-sponsored European Recovery Program (Marshall Plan). The consciousness of the importance of these American monetary aids, along with the fear of becoming a satellite of Soviet Union in case of victory of the joined Italian Communist and Socialist Parties, are some of the reasons that potentially explain the massive triumph of the Christian Democracy during April 1948 elections (women’s suffrage was granted in 1945). This was not an occasional event: from then on this party would rule the Nation for more than thirty years. In 1949 Italy joined the North Atlantic Treaty. With the restitution of the northeast-located town of Trieste and some of its neighboring territories, which were held in trust for the Allied since the end of WWII, Italy definitely acquired its actual geographical asset in 1954. The Country was admitted to join the United Nations organization at the end of 1955, and in 1957 it hosted the European Economic Community constitution summit. During this period, Italy witnessed a spectacular economic growth, characterized by an unprecedented raise of both salaries and occupational levels due to a dramatic increase in production and investments. In order to fulfill the manpower request by the industries, a massive migratory flow of work-seeking people from the south of the Country to the more developed northern regions occurred.

After the predictable depression in the scientific production witnessed by the chemical community during the wartime (Figure 4, bottom), it took some time before seeing another Italian ascending the “hall of fame”. This is literally what happened to Giulio Natta (1903–1979) whose discoveries made during the ‘50s in the field of polymerization were awarded the Nobel Prize together with Karl Ziegler in 1963. Natta graduated from the Polytechnic of Milan in 1924 and spent several years as a chemistry professor in prestigious Universities. In 1938, he was appointed Professor of General Chemistry at his *alma mater* as the application of the racial laws against Jews forced the chair’s former holder, Professor Mario Giacomo Levi, to leave. This event generated some out-of-context controversies about a potential adhesion of Natta to the Fascism [60,61], which however recently resulted in a more equilibrated judgment on the facts [62].

Natta’s work, first published in 1955 [63], led to the improvement of the organometallic-based catalysts for the low-pressure polymerization of lower alkenes earlier discovered by Ziegler [64].

**Scheme 23:**
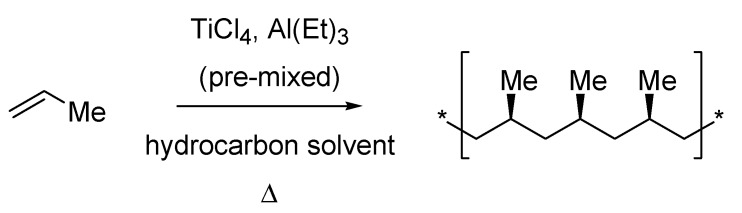
The Ziegler-Natta isotactic polypropylene synthesis (1955).

The so called Ziegler-Natta catalyst allowed the stereospecific polymerization of propylene (Scheme 23) for the first time thus revolutionizing the world of macromolecular chemistry. As the Nobel Prize committee member Arne Fredga said during the prize assignment ceremony with reference to Nature’s ability in synthesizing stereoregular polymers, “the work of Professor Natta has broken this monopoly”.

**Scheme 24:**
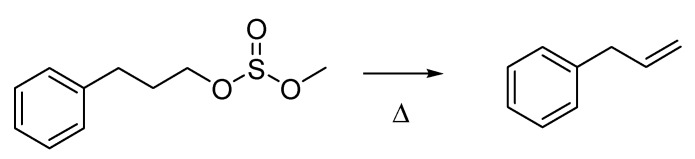
The Berti Olefination (1954).

Roughly in the same years, Giancarlo Berti (1924–2005), a young graduate from the University of Pisa, was doing his PhD at the University of Notre Dame, Indiana. He defended his dissertation entitled “The Pyrolysis of Organic Sulfites” in 1953, and gave an account of the work he had performed in a paper that was published the following year [65]. This later became known as the Berti olefination (Scheme 24), a reaction in which methyl sulfites are used to prepare unsaturated hydrocarbons. Methyl sulfites are more easily accessible than the corresponding xanthates, which are exploited in the classical Chugaev reaction [66]. In addition, their pyrolysis, although requiring slightly higher temperature than for xanthates, proceeds with higher yields thus making the Berti olefination more advantageous. 

**Scheme 25:**
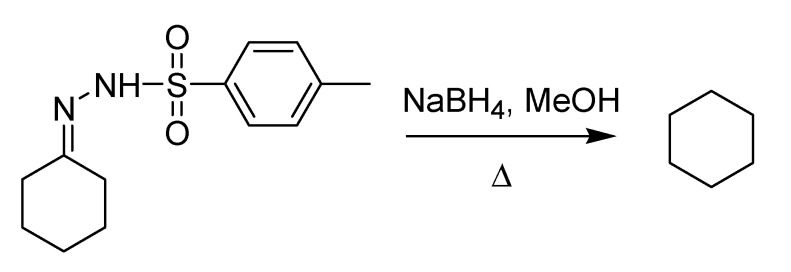
The Caglioti reduction (1964).

The publication of Berti’s paper in English was harbinger of an epochal change that was about to occur. During the subsequent decade, the habit of using English as the official language for scientific communication caught on in the Italian chemical community. Work performed at the Polytechnic of Milan represented a landmark in this respect thanks to Luciano Caglioti, who in 1964 published his first paper [67] on the reduction of tosylhdrazones to the corresponding saturated derivatives by means of NaBH_4_ (Scheme 25). The method, which is very mild and tolerates a number of functional groups, proceeds through the formation of a substituted tosylhydrazide that yields an unstable diimide, in turn decomposing to the saturated final product.

In the late ‘60s, massive movements of protest, originally mounted in US, flooded throughout Europe. Characterized by a deep socialistic connotation, these movements were highly patchy, being constituted by students, workers and poor people fighting against the inappropriateness of the political, social and educational structures of the western Nations. Originally peaceful in nature, these ideologically-driven protests rapidly became violent culminating, in Italy, with the exacerbation of the conflicts among left wing extremists, the far right and the government apparatus. Terrorists from both sides committed a number of attacks and murders aiming at destabilizing the Country. To increase the already dramatic situation, Italy experienced in the early ‘70s a profound economical crisis due to the decision of the Organization of the Arab Petroleum Exporting Countries (OAPEC) to proclaim an oil embargo. 

**Scheme 26:**
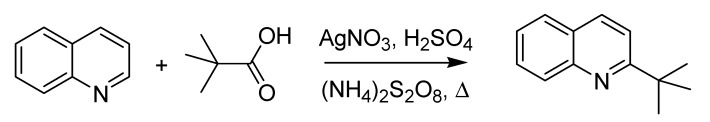
The Minisci reaction (1971).

While political radicals were acting insanely, chemical radicals were managed to react in a fruitful fashion. Thus, in 1971, a group at the Polytechnic of Milan led by Francesco Minisci, at that time still at the University of Parma, published what can be considered the first account of the so called Minisci reaction [68]. This article deals with the alkylation of heteroaromatic bases by a carbon-centered radical (Scheme 26).

An important cultural event occurred one year after the publication of Minisci’s seminal paper. After a tough ideological struggle inside the Italian Chemical Society, a decision was finally taken that all the contributions to the Gazzetta Chimica Italiana had to be written in English. 

**Scheme 27:**
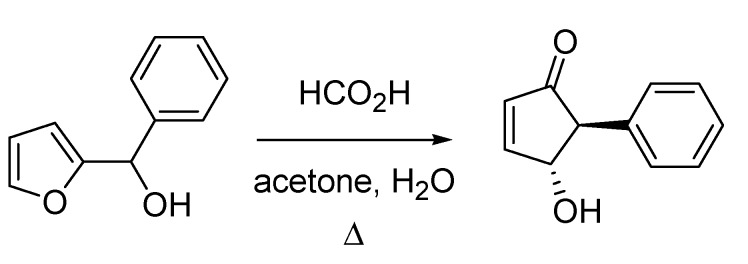
The Piancatelli rearrangement (1976).

The seventies of last century witnessed the publication of another reaction that subsequently attracted a lot of attention. This is the Piancatelli rearrangement, which was published by Giovanni Piancatelli and co-workers from the University of Rome in 1976 [69]. The reaction, which entails the synthesis of 4-hydroxycyclopent-2-enones starting from the corresponding 2-furyl carbinols, proceeds with high stereocontrol furnishing a relative *trans* orientation of the substituent at position 5 and the 4-hydroxy- group (Scheme 27). The rearrangement found some popularity as it allows accessing advanced intermediates in the synthesis of prostaglandins and other natural products.

In the meantime, the terrorist violence that was shocking Italy culminated, in March 1978, with the kidnapping of Aldo Moro, the president of the Christian Democracy party (at the time still the major party), and the assassination of his five bodyguards by a commando of the “Brigate Rosse” (Red Brigades), a left wing paramilitary organization. Despite a number of efforts by the government to safely solve the issue, Moro was killed by his kidnappers nearly two months later. However, contrary to the extremists’ expectations, this tragic event, and all the others to come up to early ‘80s, did not increase the popular adhesions to their ideologies. Furthermore, a number of important arrests beheaded the terroristic organizations which slightly dissolved alongside their utopian, and by then outdated, ideologies.

During the ‘80s Italy witnessed a tremendous economic renaissance. The Nation ranked fifth in the World’s most developed countries due to the impressive growth of the gross domestic product and to the decrease of the inflation. For the first time in its history, Italy had a socialist prime minister, Bettino Craxi, who was elected in 1983. Two years later, Michail Gorbachev became president of the Soviet Union. He was the protagonist of a number of political and social reformations that shook his country and the entire communist bloc from their very roots. One of this process most touchy outcomes was the fall of the Berlin wall (November 1989) which, aside from physically separating the eastern from the western side of the German city, was the main icon of two opposite ideologies. 

**Scheme 28:**
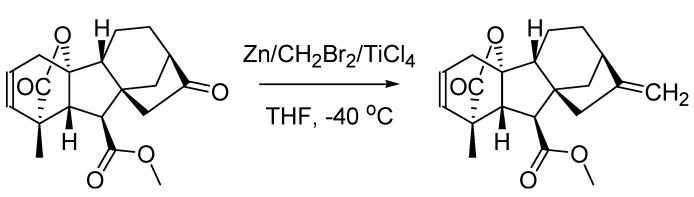
The Lombardo olefination (1982).

From a chemical standpoint, the ‘80s opened with a couple of olefination reactions. The first one was developed by Luciano Lombardo, an Italian citizen resident in Canberra, Australia, that in 1982 published the first account on the preparation and reactivity of what would later be known as the “Lombardo’s reagent” [70]. This is a highly electrophilic reagent consisting of a TiCl_4_/Zn/CH_2_Br_2_ complex, which in its original version had been disclosed by Takai’s group [71]. However, only when prepared according to the procedure reported by Lombardo this organometallic species is obtained in a very active form, enabling the mild methylenation of ketones. Noteworthy, these are not enolized by the reagent thus preventing isomerization issues (Scheme 28).

**Scheme 29:**
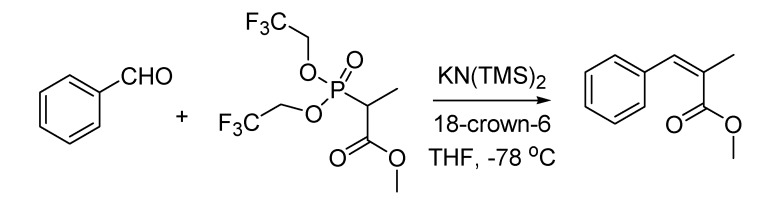
The Still-Gennari reaction (1983).

A valuable modification of the Horner-Emmons olefination reaction leading to the selective synthesis of *Z*-unsaturated esters saw the light at the Columbia University (New York, NY, USA) in 1983. Authors of the work [72] were Clark Still and Cesare Gennari, who was later to become professor at the University of Milan. The Still-Gennari reaction (Scheme 29), as this transformation is referred to nowadays, uses electrophilic bis(trifluoroethyl) phosphonoesters and a strongly dissociated base system like KN(TMS)_2_ and crown ether. The reaction is quite general in scope and it is one of the most cited methods for the synthesis of *Z*-olefins.

**Scheme 30:**
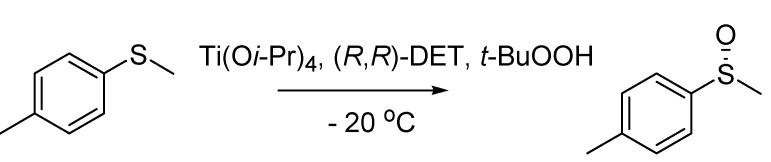
The Kagan-Modena reaction (1984).

In 1984 two groups independently published their results on the enantioselective oxidation of sulfides to sulfoxides. The first team was headed by Henri Kagan at the University of Orsay, France [73], and the second was based at the University of Padova and coordinated by Giorgio Modena [74]. The two methods are very similar and based on the Sharpless asymmetric epoxidation reaction [75], using titanium isopropoxide as the catalyst, diethyl tartrate (DET) as the chiral auxiliary, *tert*-butyl hydrogen peroxide as the oxidant. In both cases, the reaction is carried out below 0 °C. They basically differ for the solvent system employed that is dichloromethane and water in the Kagan’s method and dichloroethane in the Modena’s one. Accordingly, this oxidation procedure is generally referred to as the Kagan-Modena reaction (Scheme 30).

**Scheme 31:**
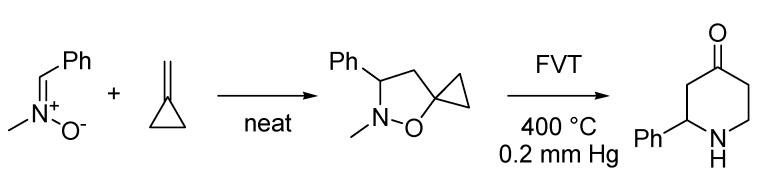
The Guarna-Brandi reaction (1986).

The Guarna-Brandi, sometimes called the Brandi reaction, consists in the formation of dihydro- or tetrahydropyridone derivatives in a one-pot two-step process involving the preparation of isoxazolines or isoxazolidines respectively, followed by their thermal rearrangement (Scheme 31). The reaction was disclosed by Antonio Guarna, Alberto Brandi and their co-workers at the University of Florence in two different moments. In its first version of 1985 [76], they exploited the cycloaddition of alkyl- or aryl-nitrile oxides with methylenecyclopropane to regioselectively synthesize isoxazolines that were thermally rearranged to 2-substituted dihydropyridine-4-ones. One year later [77] they further expanded the scope of this reaction by employing nitrones instead of nitrile oxides. The cycloaddition reaction is slightly less regioselective, but only the 5-spirocyclopropaneisoxazolidine isomer rearranges to the corresponding tetrahydropyridone. 

**Scheme 32:**
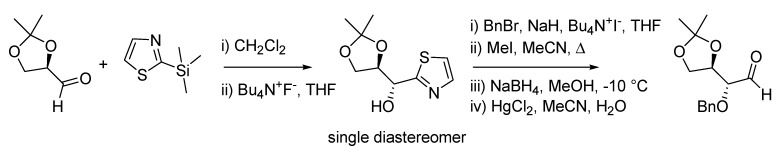
The Dondoni homologation reaction (1986).

In 1986 Alessandro Dondoni and co-workers at the University of Ferrara published an improved protocol on the use of 2-trimethylsilylthiazole in the homologation reaction of aldehydes [78], thus disclosing what is nowadays referred to as the Dondoni homologation reaction (Scheme 32). Previously, the team in Ferrara had already unveiled both the general potentialities of this reagent [79] and the aldehyde homologation sequence [80]. 

The reaction of 2-trimethylsilylthiazole (Dondoni’s reagent) with chiral aldehydes proceeds with high diasteroselectivity, and, together with the subsequent thiazole degradation sequence, delivers the homologated aldehydes in high yield. 

**Scheme 33:**
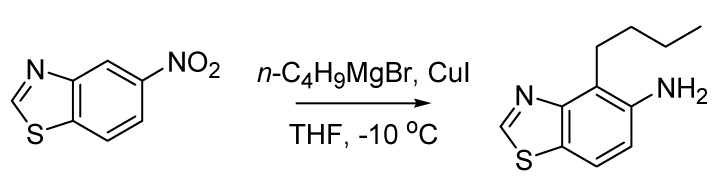
The Rosini-Bartoli reductive nitroarene alkylation (1978).

The Bartoli indole synthesis, or Bartoli reaction, appeared first in the literature in 1989, and rapidly became recognized as the most efficient means to access 7-substituted indoles [81]. In fact, the studies that culminated in the discovery of this novel reaction started back in the seventies, when a team of the University of Bologna systematically investigated nitroarenes reactivity towards organometallic compounds. This work had led to the discovery of another reaction that sometimes is referred to as the Rosini or Rosini-Bartoli reductive nitroarene alkylation, where some benzo-fused nitro derivatives are reductively alkylated in a selective manner by means of Grignard reagents. In its first version [82] this reaction allowed to prepare alkyl nitroso derivatives, however, when performed in the presence of copper (I) iodide, the reaction led to the attainment of the more useful anilines [83] (Scheme 33).

**Scheme 34:**
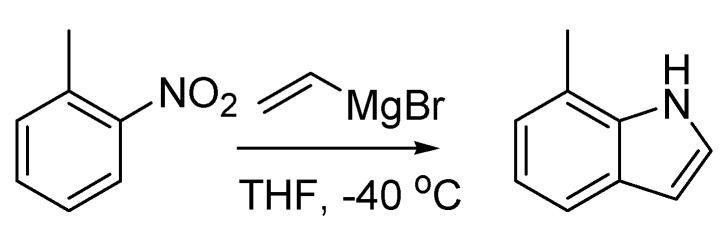
The Bartoli indole synthesis (1989).

A more popular variant of this reaction, the Bartoli indole synthesis, was discovered when an *ortho*-substituted nitroarene was reacted with a vinyl Grignard reagent. In this case the use of three moles of the organometallic species per mole of nitroarene smoothly leads to the formation of the corresponding 7-substituted indole in good yields [81] (Scheme 34).

**Scheme 35:**
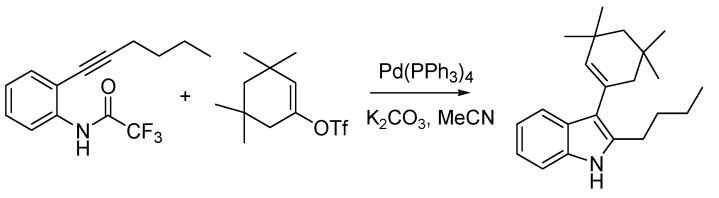
The Cacchi reaction (1992).

Indoles were also the target of the first Italian named reaction of the nineties, the Cacchi reaction (sometimes Arcadi-Cacchi reaction) [84], developed by Sandro Cacchi and co-workers at the University of Rome, providing an efficient entry into 2,3-disubstituted indoles. This is achieved starting from *o*-alkynyltrifluoroacetanilides that are reacted with vinyl triflates or aryl halides by means of a palladium-catalyzed reaction (Scheme 35).

**Scheme 36:**
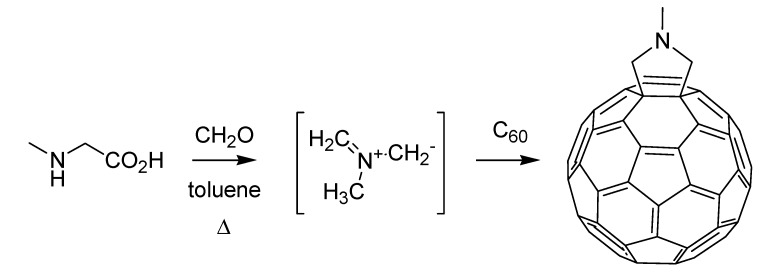
The Prato reaction (1993).

Palladium chemistry was one of the workhorses of organic synthesis during the nineties, and besides the Cacchi reaction a further important contribution from Italian scientists would appear during the decade (*vide infra*). Before that, however, another hot topic of chemical research of the time witnessed the publication of a seminal paper by an Italian group. This is the chemistry of fullerenes, in which Maurizio Prato of the University of Trieste and his colleagues at Padua left their mark [85] thanks to the engagement of C_60_ in a 1,3-dipolar cycloaddition reaction, using azomethine ylides generated *in situ*, resulting in the corresponding fulleropyrrolidines (Scheme 36).

In 1992, Italy was shocked by an unprecedented scandal that, starting from Milan, rapidly ran over the whole country. A number of kickbacks were discovered to be cashed in by the politicians in exchange for contracts. The politicians, in their turn, deposited part of the illegal incomes in their parties’ accounts. The scandal that initially involved only minor characters within the parties, finally reached their summits, with a number of parties’ secretaries recognized to be involved. The party which paid the highest price was the Socialist one whose leader and former Prime Minister Craxi resigned and left Italy for Tunisia, where he would die in 2000. 

None of the economical sectors was exempted from the corruption: the pharmaceutical industry, by illegally financing the health minister and his managers for having the price of their drugs increased or falsely recognized as indispensable; the chemical industry, for the enormous amount of kickbacks linked to certain public and private companies’ joint ventures maneuvers. 

This scandal, however, had the merit to unearth the corrupted connections between the unscrupulous component of the private sector and part of the public institutions. The moralization wind which followed originated what was colloquially called the “Second Republic”. By the nomination of high-profile rulers (e.g., the Prime Minister Carlo Azeglio Ciampi, elected in 1993, who would later become the 10 President of the Republic), the government tried to distance from the maladministration of the past and to regain the trust of the citizens. Between 1994 and 1997, a center-right alliance, a technical government and finally a center-left coalition succeeded in ruling the Nation [86].

**Scheme 37:**
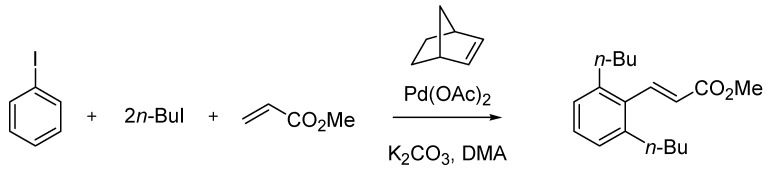
The Catellani reaction (1997).

In the twilight of the century, when the Italian chemical and pharmaceutical industry was still suffering the repercussions of the scandal that had gripped it a few years before, a highly significant reaction belonging to the universe of palladium-mediated organic synthesis was published by Marta Catellani and co-workers [87] at the University of Parma. The Catellani reaction provides a very elegant entry into the synthesis of *o*,*o*-disubstituted vinylarenes starting from aryl iodides. The reaction exploits a multicomponent protocol where, together with the reactants and catalyst, norbornene or another strained olefin is used. The latter is essential as it enters the complex catalytic cycle by activating three adjacent positions of the arene, being recycled at the end of the process (Scheme 37). 

**Figure 5:**
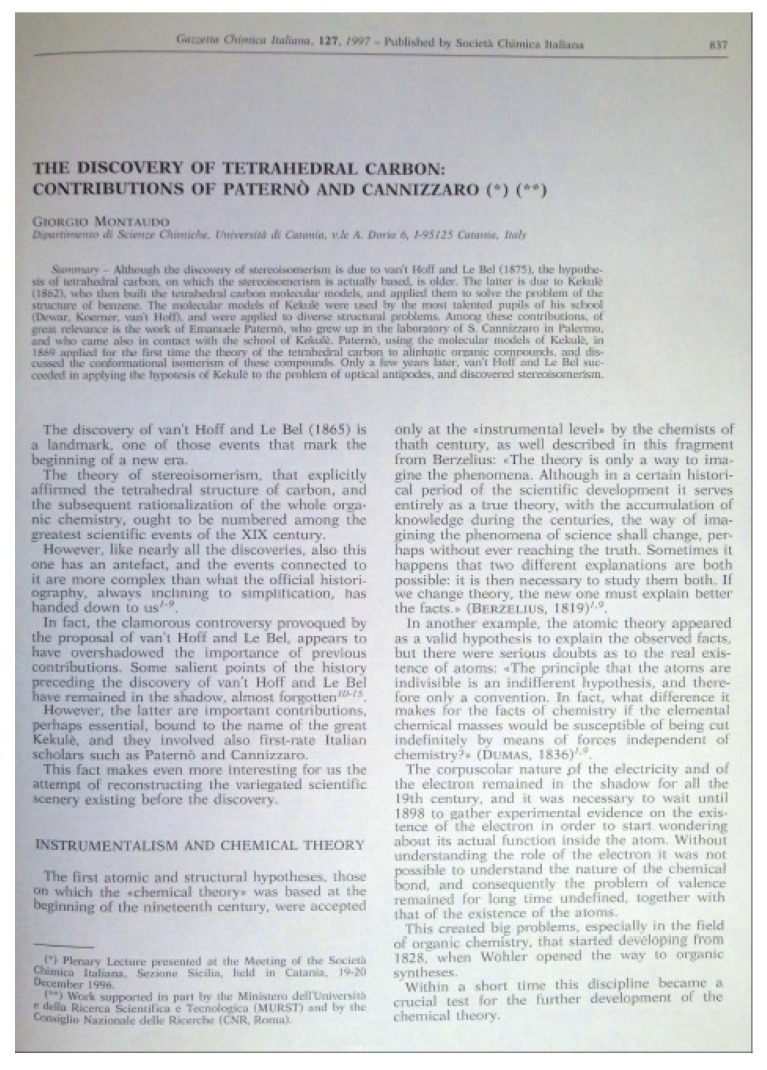
The first page of the last article published in the Gazzetta Chimica Italiana (**1997**, *127*, 837–842) (from the Nerviano Medical Sciences library).

In December 1997, the Gazzetta Chimica Italiana published its last issue, thus ceasing its activity after 127 years of glorious history. Since 1998, in fact, most of the European Societies’ journals have flowed into the two newly-founded European Journal of Organic Chemistry and European Journal of Inorganic Chemistry [88]. The last article of the Gazzetta, authored by Giorgio Montaudo, dealt with the contribution of Paternò and Cannizzaro (two of the founding members of the journal) to the discovery of the tetrahedral carbon (Figure 5). It was an elegant way of closing the circle.

Traveling back and forth through the animated landscape of about 150 years of Italian history, we have finally reached the twenty-first century, where our tale finishes. The present day is too close to be recounted with adequate objectivity, just as for current politics, as for newly discovered organic reactions. They are either still in the need to pass the test of time or simply awaiting for someone to christen them according to their inventors’ names.

## 7. Conclusions

We have discussed the contribution to the discovery of new chemical reactions by Italian scientists, whose names have been associated to these reactions. By walking through almost 150 years of Italian history, we have also aimed at contextualizing these discoveries within the brighter and darker moments of the country’s history. Indeed, because of the historical in nature of this review, reactants and reaction conditions shown in the schemes were, whenever possible, taken from the original articles. Readers interested in the topic are encouraged to refer to recent literature for the developments, scopes and limitations of each chemical transformation reported. Finally, as stated in the introductory section, the “named organic reactions” chemical jargon is far from being undisputedly objective. Thus, we apologize to the chemists whose contributions may have involuntarily been omitted from this review. Oversights, misrecognitions and misattributions in science are fortunately infrequent but, being science a human act, they cannot be completely eradicated. 

## References

[1] Hassner A., Namboothiri I. (2011). Organic Synthesis Based on Name Reactions.

[2] Li J.J. (2009). Name Reactions: A Collection of Detailed Reaction Mechanisms.

[3] Kürti L., Czakó B. (2005). Strategic Applications of Named Reactions in Organic Synthesis.

[4] Wang Z. (2009). Comprehensive Organic Name Reactions and Reagents.

[5] Gal J. (2012). The discovery of stereoselectivity at biological receptors: Arnaldo Piutti and the taste of the asparagine enantiomers—History and analysis on the 125th anniversary. Chirality.

[6] Cannizzaro S., Piria E. (1883). Sulla vita e sulle opere di Raffaele.

[7] Piria R. (1846). Studi sulla costituzione chimica dell’asparagina e dell’acido aspartico. Il Cimento.

[8] Piria R. (1850). Sull’azione del solfito d’ammoniaca sulla nitronaftalina e i suoi prodotti che da quella derivano.

[9] Piria R. (1851). Ueber einige Produkte Einwirkung des schwefligsauren Ammoniaks auf Nitrophtalin. Liebigs Ann..

[B10-molecules-18-10870] 10.Some Italian organic chemistry textbooks (*i.e.*, Fusco, R.; Bianchetti, G.; Rosnati, V. *Chimica Organica Volume 1* (Organic Chemistry Volume 1), 2nd ed.; CEA: Milano, 1974) refer to the “Piria reaction” to indicate the synthesis of aldehydes from the corresponding carboxylic acids by heating the latter in the presence of calcium formate and, by extension, the thermal decarboxylative coupling of calcium salts of carboxylic acid to form the corresponding simmetric ketones. These reactions were indeed first performed by Piria, who described the method in a letter to S. Cannizzaro dated 26 June 1854, where he acknowledge “some experience on ketones” by Williamson, who had predicted the transformation but never performed it (Williamson, A. Ueber aetherbildung (On ethers formation). *Liebigs Ann.* **1852**, *81*, 73–78.

[11] Bertagnini C. (1851). Sulle combinazioni di alcuni oli essenziali con i solfiti alcalini. Annali delle università toscane.

[12] Bertagnini C. (1853). Ueber die verbindungen einiger flüchtigen oele mit den zweifach-schwefligsauren alkaline. Liebigs Ann..

[13] Bertagnini C. (1853). Ueber die verbindungen einiger flüchtigen oele mit den zweifach-schwefligsauren alkaline. Liebigs Ann..

[14] Perkin W.H. (1868). On the hydride of aceto-salicyl. J. Chem. Soc..

[15] Bertagnini C. (1856). Produzione artificiale dell’acido cinnamico, ottenuto per lungo riscaldamento a 120°–130° di aldeide benzoica e cloruro di acetile anidri. Nuovo Cimento.

[16] Bertagnini C. (1856). Ueber die künstliche darstellung der zimmtsäure. Liebigs Ann..

[17] Chiozza L. (1856). Ueber die künstliche bildung des cinnamylwasserstoffs. Liebigs Ann..

[18] Cannizzaro S. (1853). Ueber den der benzoësäure entsprechenden alkohol. Liebigs Ann..

[19] Cannizzaro S. (1991). Sunto di un corso di filosofia chimica.

[20] Schiff H. (1864). Eine neue reihe organischer basen. Liebigs Ann..

[21] Schiff H. (1866). Eine neue reihe organischer diamine. Liebigs Ann..

[22] Ciamician G.L., Dennstedt M. (1881). Sull’azione del cloroformio sul composto potassico del pirolo. Gazz. Chim. Ital..

[23] Ciamician G.L., Dennstedt M. (1881). Ueber die einwirkung des chloroforms auf die kaliumverbindung pyrrols. Berichte der deutschen chemischen Gesellschaft.

[24] Ciamician G., Silber P. (1900). Chemische Lichtwirkungen. Berichte der deutschen chemischen Gesellschaft.

[25] Schönberg A. (1968). Preparative Organic Photochemistry.

[26] Ciamician G., Silber P. (1908). Chemische Lichtwirkungen. Berichte der deutschen chemischen Gesellschaft.

[27] Enciclopedia italiana Treccani. http://www.treccani.it/enciclopedia/giacomo-ciamician_(Dizionario-Biografico)/.

[28] Ciamician G., Silber P. (1901). Chemische Lichtwirkungen (Chemical effects of light). Berichte der deutschen chemischen Gesellschaft.

[29] La Fotochimica Dell’avvenire.

[30] Plancher G. II. (1898). Ricerche sull’azione dei ioduri alcolici sugli indoli. Sulla β-etil-β-N-dimetil-α-metilindolina. Gazz. Chim. Ital..

[31] Biginelli P. (1891). Intorno ad uramidi aldeidiche dell’etere acetilacetico. Gazz. Chim. Ital..

[32] Biginelli P. (1891). Intorno ad uramidi aldeidiche dell’etere acetilacetico. II. Gazz. Chim. Ital..

[33] Biginelli P. (1924). Used for the detection of picric acid in biological liquids. Ann. Chim. Appl..

[34] Tron G.C., Minassi A., Appendino G. (2011). Pietro Biginelli: The man behind the reaction. Eur. J. Org. Chem..

[B35-molecules-18-10870] 35.Originally, he collaborated with professor Selmi to the compilation of the “Enciclopedia di Chimica scientifica e industriale” (Scientific and Industrial Chemistry Encyclopedia), whose publication commenced in 1868. Later he edited his own encyclopedia: “Nuova enciclopedia di chimica scientifica, tecnologica e industriale diretta dal prof. Icilio Guareschi con la collaborazione di distinti chimici italiani” (New Scientific, Technological and Industrial Chemistry Encyclopedia edited by Professor Icilio Guareschi with the collaboration of distinguished Italian chemists), Torino: Unione Tipografico Editrice, 1915.

[36] Guareschi I. (1896). Sintesi di composti piridinici dagli eteri chetonici coll’etere cianacetico in presenza dell’ammoniaca e delle amine. Mem. Reale Accad. Sci. Torino.

[37] Thole F.B., Thorpe J.F. (1911). The formation and reactions of iminocompounds. Part XV. The production of imino-derivatives of piperidine leading to the formation of the ββ-disubstituted glutaric acids. J. Chem. Soc. Trans..

[38] Ponzio G. (1897). Azione del tetrossido d’azoto sugli isonitrosochetoni. Gazz. Chim. Ital..

[39] Pellizzari G. (1894). Nuova sintesi del triazolo e dei suoi derivati. Gazz. Chim. Ital..

[40] Ortoleva G. (1899). Azione del jodio sull’acido cinnamico in soluzione piritica. Gazz. Chim. Ital..

[41] Ortoleva G. (1900). Azione del jodio sull’acido malonico in soluzione piridica. Gazz. Chim. Ital..

[42] King L.C. (1944). The reaction of iodine with some ketones in the presence of pyridine. J. Am. Chem. Soc..

[43] Betti M. (1900). Sull’addizione di basi aldeido-aminiche ai naftoli. Gazz. Chim. Ital..

[44] Paolocci N., Wink D.A. (2009). The shy Angeli and his elusive creature: The HNO route to vasodilatation. Am. J. Physiol. Heart Circ. Physiol..

[45] Angeli A. (1896). Sopra la nitroidrossilammina. Gazz. Chim. Ital..

[46] Angeli A., Angelico F. (1900). Sopra l’acido nitroidrossilamminico. Gazz. Chim. Ital..

[47] Rimini E. (1901). Sopra una nuova reazione delle aldeidi. Gazz. Chim. Ital..

[48] Young E.G., Conway C.F. (1942). On the estimation of allantoin by the Rimini-Schryver reaction. J. Biol. Chem..

[49] Rimini E. (1898). Sul riconoscimento della formaldeide negli alimenti. Ann. di Farmacot. e Chim..

[50] Schryver S.B. (1910). The photochemical formation of formaldehyde in green plants. Proc. R. Soc. Lond. Ser. B.

[51] Link G. Verfahren zur Darstellung von Oxy-i-butyryl-Phenolen. German Patent.

[52] Bargellini G. (1906). Azione del cloroformio e idrato sodico sui fenoli in soluzione nell’acetone. Gazz. Chim. Ital..

[53] Paternò E., Chieffi G. (1909). Sintesi in chimica organica per mezzo della luce. Nota II. Composti degli idrocarburi non saturi con aldeidi e chetoni. Gazz. Chim. Ital..

[54] Büchi G., Inman C.G., Lipinsky E.S. (1954). Light-catalyzed Organic Reactions. I. The reaction of carbonyl compounds with 2-methyl-2-butene in the presence of ultraviolet light. J. Am. Chem. Soc..

[55] Gastaldi C. (1921). Sulle pirazine (On pyrazines). Gazz. Chim. Ital..

[56] Passerini M. (1921). Sopra gli isonitrili (I). Composto del *p*-isonitril-azobenzolo con acetone ed acido acetico. Gazz. Chim. Ital..

[57] Passerini M. (1921). Sopra gli isonitrili (II). Composti con aldeidi o con chetoni ad acidi organici monobasici. Gazz. Chim. Ital..

[58] Amadori M. (1925). Prodotti di condensazione tra il glucosio e la para fenetidina. Parte I. Atti della Accademia Nazionale dei Lincei, Classe di Scienze Fisiche, Matematiche e Naturali, Rendiconti.

[59] Mascarelli L. (1936). Contributo alla conoscenza del bifenile e dei suoi derivati.—Nota XV. Passaggio dal sistema bifenilico a quello fluorenico. Gazz. Chim. Ital..

[60] Hargittai I., Comotti A., Hargittai M. (2003). Giulio Natta. Chem. Eng. News.

[61] Hargittai I., Hargittai M. (2003). Giulio Natta: A complex portrait. Chem. Eng. News.

[62] Girelli A.  (2003). I chimici e il fascismo. La Chimica e l’Industria.

[63] Natta G. (1955). Una nuova classe di polimeri di alfa-olefine aventi una eccezionale regolarità di strutture. Atti Acc. Naz. Lincei Mem..

[64] Ziegler K. (1952). Neuartige katalytische Umwandlungen von Olefinen. Brennst. Chem..

[65] Berti G. (1954). The Pyrolysis of Sulfites. III. Methyl Alkyl Sulfites. A new method for the preparation of olefins. J. Am. Chem. Soc..

[66] Chugaev L. (1899). Uber eine neue methode aur darstellung ungesättigter kohlenwasserstoffe. Berichte der deutschen chemischen Gesellschaft.

[67] Caglioti L., Grasselli P. (1964). A new method for the reduction of aldehydes and ketones to CH_2_ under mild conditions. Chem. Ind. Lond..

[68] Minisci F., Bernardi R., Bestini F., Galli R., Perchinummo M. (1971). Nucleophilic character of alkyl radicals-VI: A new convenient selective alkylation of heteroaromatic bases. Tetrahedron.

[69] Piancatelli G., Scettri A., Barbadoro S. (1976). A useful preparation of 4-substituted 5-hydroxy-3-oxocyclopentene. Tetrahedron Lett..

[70] Lombardo L. (1982). Methylenation of carbonyl compounds with Zn-CH_2_Br_2_-TiCl_4_. Applications to gibberellins. Tetrahedron Lett..

[71] Takai K., Hotta Y., Oshima K., Nozaki H. (1978). Effective methods of carbonyl methylenation using CH_2_I_2_-Zn-Me_3_Al and CH_2_Br_2_-Zn-TiCl_4_ system. Tetrahedron Lett..

[72] Still W.C., Gennari C. (1983). Direct synthesis of Z-unsaturated esters. A useful modification of the Horner-Emmons olefination. Tetrahedron Lett..

[73] Pitchen P., Kagan H.B. (1984). An efficient asymmetric oxidation of sulfides to sulfoxides. Tetrahedron Lett..

[74] Di Furia F., Modena G., Seraglia R. (1984). Synthesis of chiral sulfoxides by metal-catalyzed oxidation with *t*-butyl hydroperoxide. Synthesis.

[75] Katsuki T., Sharpless K.B. (1980). The first practical method for asymmetric epoxidation. J. Am. Chem. Soc..

[76] Guarna A., Brandi A., Goti A., de Sarlo F. (1985). 4,5-dihydroisoxazole-5-spirocyclopropanes. Synthesis and thermolytic rearrangement to 5,6-dihydro-4-pyridones. J. Chem. Soc. Chem. Commun..

[77] Brandi A., Guarna A., Goti A., de Sarlo F. (1986). Rearrangement of nitrose cycloadducts to methylene cyclopropane. Synthesis of indolizidine and quinolizidine derivatives. Tetrahedron Lett..

[78] Dondoni A., Fantin G., Fogagnolo M., Medici A. (1986). Synthesis of long-chain sugars by iterative, diastereoselective homologation of 2,3-*o*-isopropylidene-d-glyceraldehyde with 2-trimethylsilylthiazole. Angew. Chem. Int. Ed..

[79] Medici A., Fantin G., Fogagnolo M., Dondoni A. (1983). Reactions of 2-trimethylsilylthiazole with acyl chlorides and aldehydes synthesis of new thiazol-2-yl derivatives. Tetrahedron Lett..

[80] Dondoni A., Fogagnolo M., Medici A. Pedrini (1985). Diastereoselectivity in the 1,2-addition of silylazoles to chiral aldehydes. Stereocontrolled homologation of α-hydroxyaldehydes. Tetrahedron Lett..

[81] Bartoli G., Palmieri G., Bosco M., Dalpozzo R. (1989). The reaction of vinyl Grignard reagents with 2-substituted nitroarenes: A new approach to the synthesis of 7-substituted indoles. Tetrahedron Lett..

[82] Bartoli G., Rosini G. (1976). Reductive alkylation of 6-nitrobenzothiazoles with Grignard reagents: Synthesis of 7-alkyl-6-nitrosobenzothiazoles. Synthesis.

[83] Bartoli G., Medici A., Rosini G., Tavernari D. (1978). Reductive C-Alkylation of nitroarenes with Grignard reagents; Synthesis of alkyl-amino-arenes. Synthesis.

[84] Arcadi A., Cacchi S., Marinelli F. (1992). A versatile approach to 2,3-disubstituted indoles through the palladium-catalysed cyclization of *o*-alkynyltrifluoroacetanilides with vinyl triflates and aryl halides. Tetrahedron Lett..

[85] Maggini M., Scorrano G., Prato M. (1993). Addition of azomethine ylides to C_60_: Synthesis, characterization, and functionalization of fullerene pyrrolidines. J. Am. Chem. Soc..

[86] (2004). The vast majority of the historical information reported in this review has been taken from: Montanelli, I. Storia d’Italia.

[87] Catellani M., Frignani F., Rangoni A. (1997). A complex catalytic cycle leading to a regioselective synthesis of *o*,*o′*-disubstituted vinylarenes. Angew. Chem. Int. Ed..

[88] Gennari C., Peruzzini M. (2009). The Italian chemical society is 100 years old. Eur. J. Org. Chem..

